# Long Term Outcomes Following Hospital Admission for Sepsis Using Relative Survival Analysis: A Prospective Cohort Study of 1,092 Patients with 5 Year Follow Up

**DOI:** 10.1371/journal.pone.0112224

**Published:** 2014-12-08

**Authors:** Joshua S. Davis, Vincent He, Nicholas M. Anstey, John R. Condon

**Affiliations:** 1 Menzies School of Health Research, Charles Darwin University, Darwin, Australia; 2 Department of Infectious Diseases, Royal Darwin Hospital, Darwin, Australia; University of Pittsburgh, United States of America

## Abstract

**Background:**

Sepsis is a leading cause of death in intensive care units and is increasing in incidence. Current trials of novel therapeutic approaches for sepsis focus on 28-day mortality as the primary outcome measure, but excess mortality may extend well beyond this time period.

**Methods:**

We used relative survival analysis to examine excess mortality in a cohort of 1,028 patients admitted to a tertiary referral hospital with sepsis during 2007–2008, over the first 5 years of follow up. Expected survival was estimated using the Ederer II method, using Australian life tables as the reference population. Cumulative and interval specific relative survival were estimated by age group, sex, sepsis severity and Indigenous status.

**Results:**

Patients were followed for a median of 4.5 years (range 0–5.2). Of the 1028 patients, the mean age was 46.9 years, 52% were male, 228 (22.2%) had severe sepsis and 218 (21%) died during the follow up period. Mortality based on cumulative relative survival exceeded that of the reference population for the first 2 years post admission in the whole cohort and for the first 3 years in the subgroup with severe sepsis. Independent predictors of mortality over the whole follow up period were male sex, Indigenous Australian ethnicity, older age, higher Charlson Comorbidity Index, and sepsis-related organ dysfunction at presentation.

**Conclusions:**

The mortality rate of patients hospitalised with sepsis exceeds that of the general population until 2 years post admission. Efforts to improve outcomes from sepsis should examine longer term outcomes than the traditional primary endpoints of 28-day and 90-day mortality.

## Introduction

Severe sepsis is the most common cause of death in intensive care units [1], and its incidence has progressively increased over the past 20 years [2], [3]. Until recently, most clinical trials of new therapeutic approaches for severe sepsis have used 28-day mortality as their primary endpoint [4]–[6]. However, it is now increasingly recognised that the sequelae of sepsis extend well beyond the index hospitalisation and that longer-term outcomes should be used in order to better understand the effect of a given intervention [7], [8]. Despite this increased recent interest, it remains unclear how long the excess mortality risk persists after an episode of severe sepsis, with estimates ranging from 90 days to 5 years [8]–[13].

Most existing studies investigating longer-term outcomes of sepsis are difficult to extrapolate for several reasons. The majority of sepsis outcome studies include only patients admitted to an intensive care unit (ICU), but only 50–70% of patients hospitalised with severe sepsis ever enter an ICU [14], [15]. Most of these studies use retrospective analysis of existing large datasets, potentially underestimating the true incidence of sepsis [16]. Most importantly, with rare exceptions [13], these studies generally do not compare sepsis outcomes with those of an appropriately matched general population.

Relative survival analysis is a statistical technique commonly used in oncology [17]–[19], but it has very rarely been applied to sepsis outcomes [20]. It compares the survival of a cohort of patients over time with that of a background reference population.

We aimed to describe the long term outcomes of a prospectively recruited cohort of patients with sepsis, including those admitted to ICU and non-ICU wards. Our primary aim was to estimate the duration of the excess mortality risk in patients with sepsis over the first 5 years of follow up, using relative survival analysis.

## Methods

### Patients and Setting

The patients included in this cohort have been previously described in detail [21]. In brief, we prospectively enrolled every patient admitted over a 365 day period in 2007–2008 who met the 1992 ACCP-SCCM criteria for sepsis [22], in a tertiary referral hospital in tropical Australia.

Where patients were admitted with more than one episode of sepsis over the 12-month course of the original study, only the first episode was included in the current analysis. Furthermore, all patients who were resident outside the Northern Territory in the year of initial admission (n = 62) were excluded from the current analysis, because their vital status was difficult to determine accurately in the longer term.

### Ethics approval

This study was approved by the Human Research Ethics Committee of the Menzies School of Health Research and Northern Territory Department of Health, who waived the requirement for individual informed consent.

### Definitions

Severe sepsis was defined as sepsis plus at least one attributable organ dysfunction within the previous 24 hours, as per the definitions used in the PROWESS study [4]. Comorbidities were as defined by Charlson et al and quantified using the Charlson Comorbidity Index [23].

### Outcomes

Information about all deaths that occur in the Northern Territory is provided to the Northern Territory Department of Health by the Registrar of Births, Deaths and Marriages and recorded in the public hospitals' client administration system. We accessed this deaths information to determine vital status for all patients included in this study at 12 months, 3 years and 5 years after the commencement of the study.

### Survival analysis

Patients were included in the survival model until death from any cause, censoring due to loss to follow up, or the end of the follow-up period (30/06/2012). Mortality of sepsis patients was measured as both overall survival and relative survival. Overall survival (also referred to as crude survival) is the proportion of sepsis patients still alive at a certain point in time after their sepsis episode; deaths include those unrelated to the sepsis episode, so overall survival does not measure excess mortality related specifically to the sepsis episode. Overall survival analysis was calculated using the Kaplan-Meier method.

By comparing deaths among the sepsis patients with the expected number of deaths based on general population mortality rates, relative survival is an estimate of excess mortality in the sepsis patients. To estimate relative survival, the background mortality rate for the general population was derived from life tables. Expected survival was estimated using the Ederer II method [24] from Australian population life tables for non-Indigenous people and life tables for the total NT Indigenous population for Indigenous people, stratified by age, sex and calendar period. At the time of analysis, life table data were only available up until 2006, therefore, expected survival for the study period were based on 2006 data. The results of relative survival analysis were expressed in several ways: one-year and five-year relative survival; interval-specific relative survival; and the excess mortality rate. Cumulative relative survival is the observed survival among the subjects under study at a point in time after their first sepsis episode divided by the expected survival of people of the same age, sex and Indigenous status in the general population; for example, five-year relative survival is the cumulative relative survival at five years after first sepsis episode [24]. In general, cumulative relative survival progressively decreases over time since the first episode until there is no more attributable excess mortality of the condition of interest (relative to the background population), after which it plateaus at a constant level. Interval-specific relative survival is the ratio of the survival in the population of interest relative to the general population during specific time intervals. Interval-specific relative survival is lower than 1.0 when the survival of the study cohort is lower than expected (based on the mortality rates of the general population) and plateaus at 1.0 when the survival of the cohort becomes the same as the background population. The excess mortality rate is the difference between the observed mortality rate in the subjects and the expected rate based on that of the general population(matched for age, sex and Indigenous status) [25].

### Statistical analysis

All analyses were conducted using Stata/SE 12 (StataCorp, College Station, TX). Continuous variables were compared using t-test for normally distributed data and Mann-Whitney U test for non-normal data. Categorical variables were compared using χ^2^ test. Crude survival rates were derived using Kaplan-Meier estimates. Interval-specific relative survival ratio (RSR) and cumulative RSR were derived using the Stata user-defined program “strs” [26] and excess mortality rates were derived using flexible parametric models, fitted using the Stata user-defined program “stpm2” [25].

A generalized linear model with Poisson error structure was used to model the excess mortality based on collapsed data, in which the outcome was the observed number of deaths and was assumed to be Poisson distributed. The predictors of excess mortality were chosen using forward selection, resulting in the final model with the following covariates: follow-up time since diagnosis, gender, Indigenous status, age group, Charlson index group, severe sepsis and bacteraemic.

## Results

### Patient demographics and outcomes

Patients were followed for a median of 4.5 years (range 0–5.2 years), giving a cumulative time at risk of 3,997 patient-years. Of the 1,028 patients with sepsis, 218 had died by the end of the follow-up period. Severe sepsis was present in 228 patients (22% - **Table 1**). Those who died during the follow up period were older, more likely to be male and Indigenous, had greater sepsis severity at baseline and a larger number of co-morbidities. Pneumonia was over-represented amongst those who died, and skin and soft tissue infection was under-represented (**Table 1**).

**Table 1:** Demographics, comorbidities, characteristics of infection and disease severity in those who died compared with those who were alive at the end of follow up.

	Total (n = 1,028)	Died (n = 218)	Alive (n = 810)	P value^a^
**Predisposition**				
Age (years)^b^	46.9 (17.3)	58.9 (15.7)	43.6 (16.2)	<0.001
Male	532 (51.8%)	128 (58.7%)	404 (49.9%)	0.02
Indigenous	510 (49.6%)	128 (58.7%)	382 (47.2%)	<0.001
Hazardous alcohol use^c^	284/636 (44.7%)	70/133 (52.6%)	214/503 (42.5%)	0.04
Current smoking^c^	329/694 (47.4%)	69/139 (49.6%)	296/555 (53.3%)	0.44
Chronic renal disease	122 (11.9%)	54 (24.8%)	68 (8.4%)	<0.001
Chronic liver disease	79 (7.7%)	32 (14.7%)	47 (5.8%)	<0.001
Diabetes	250 (24.3%)	81 (37.2%)	169 (20.9%)	<0.001
COPD	135 (13.1%)	54 (24.8%)	81 (10.0%)	<0.001
Malignancy	44 (4.3%)	22 (10.1%)	22 (2.7%)	<0.001
Charlson comorbidity index^d^	1 (0–2)	3 (1–4)	0 (0–1)	<0.001
**Infection**				
Bacteraemic	163 (15.8%)	61 (28.0%)	102 (12.6%)	<0.001
Pneumonia	317 (30.8%)	96 (44.0%)	221 (27.3%)	<0.001
Urosepsis	122 (11.9%)	34 (15.6%)	88 (10.9%)	0.06
Skin and soft tissue infection	349 (34.0%)	43 (19.7%)	306 (37.8%)	<0.001
Intra-abdomnal infection	109 (10.6%)	17 (7.8%)	92 (11.4%)	0.13
Gram positive bacterium	255 (24.8%)	50 (22.9%)	205 (25.3%)	0.47
Gram negative bacterium	205 (19.9%)	50 (22.9%)	155 (19.1%)	0.21
**Response and Organ Dysfunction**				
Required ICU admission	161 (15.7%)	62 (28.4%)	99 (12.2%)	<0.001
Severe sepsis	228 (22.2%)	77 (35.3%)	151 (18.6%)	<0.001
APACHE II score^d^	8 (4–13)	14 (9–20)	6 (3–10)	<0.001
SOFA score^d^	1 (0–3)	3 (1–5)	1 (0–2)	<0.001

Data are given as n(%) unless stated otherwise.

Parameters are grouped according to PIRO classification [39].

a P-value comparing those alive at end of follow up period compared with those who were not.

b Mean (SD).

c Data were not available for all patients regarding alcohol use and smoking.

d Median (IQR).

### Crude survival analysis

One-year and five-year crude survival for all sepsis patients (with 95% confidence intervals) were 87.5% (85.3%–89.3%), and 77.6%(74.5%–80.4%) respectively. The corresponding figures for the severe sepsis subgroup were 74.1% (67.9%–79.3%) and 66.2% (59.7%–72.0%).

**Figure 1:**
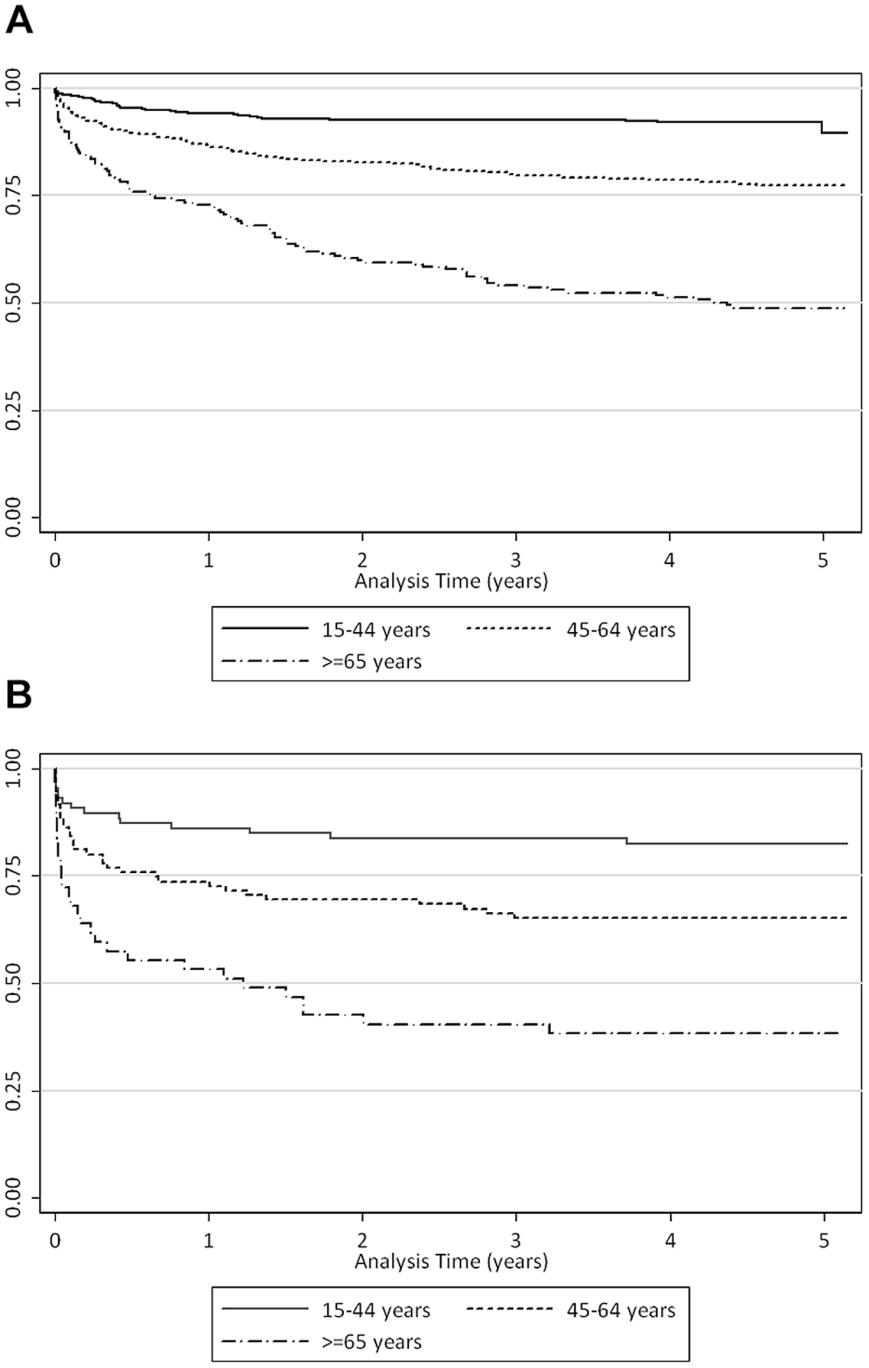
a. Kaplan-Meier crude survival estimates by age group for all sepsis patients (n = 1,028). b. Kaplan-Meier crude survival estimates by age group for severe sepsis patients (n = 228).

Kaplan-Meier estimates by age group are shown in **Fig. 1**, in which people aged over 64 years had the lowest crude survival and people under 45 years old had the highest.

### Relative survival analysis

**Figure 2:**
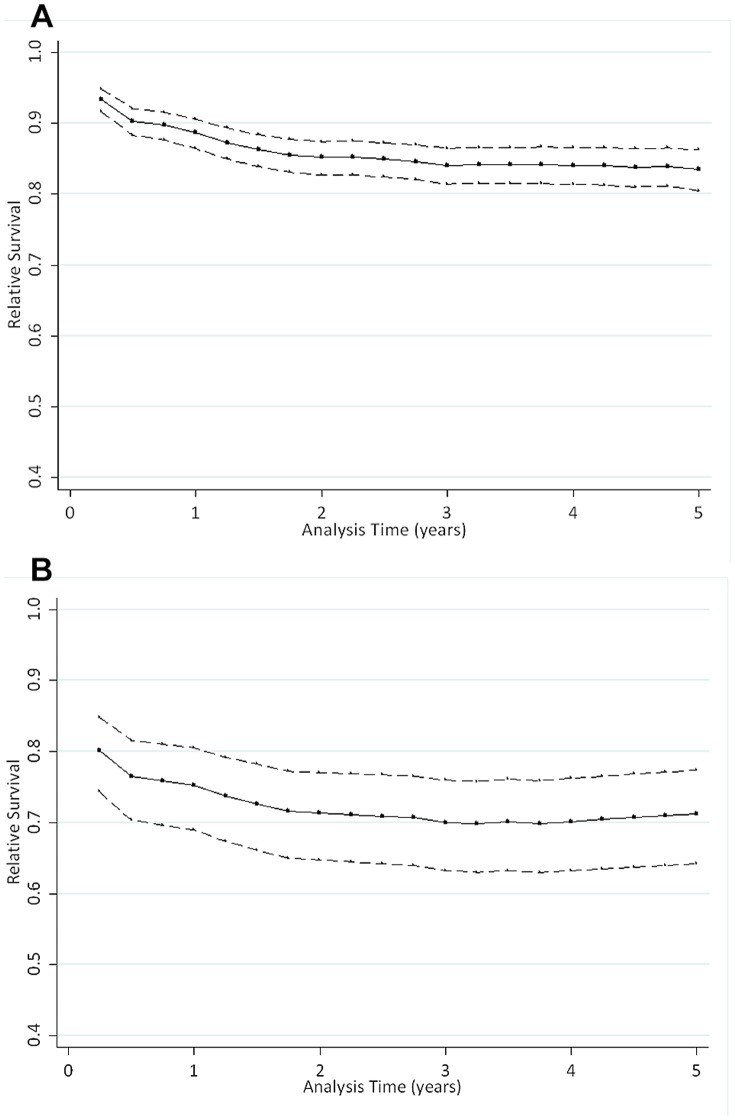
a. Cumulative relative survival for patients with sepsis and severe sepsis, compared with a reference population. Dotted lines represent 95% confidence intervals. b. Cumulative relative survival for only patients with severe sepsis (n = 228), compared with a reference population. Dotted lines represent 95% confidence intervals.

**Figure 3:**
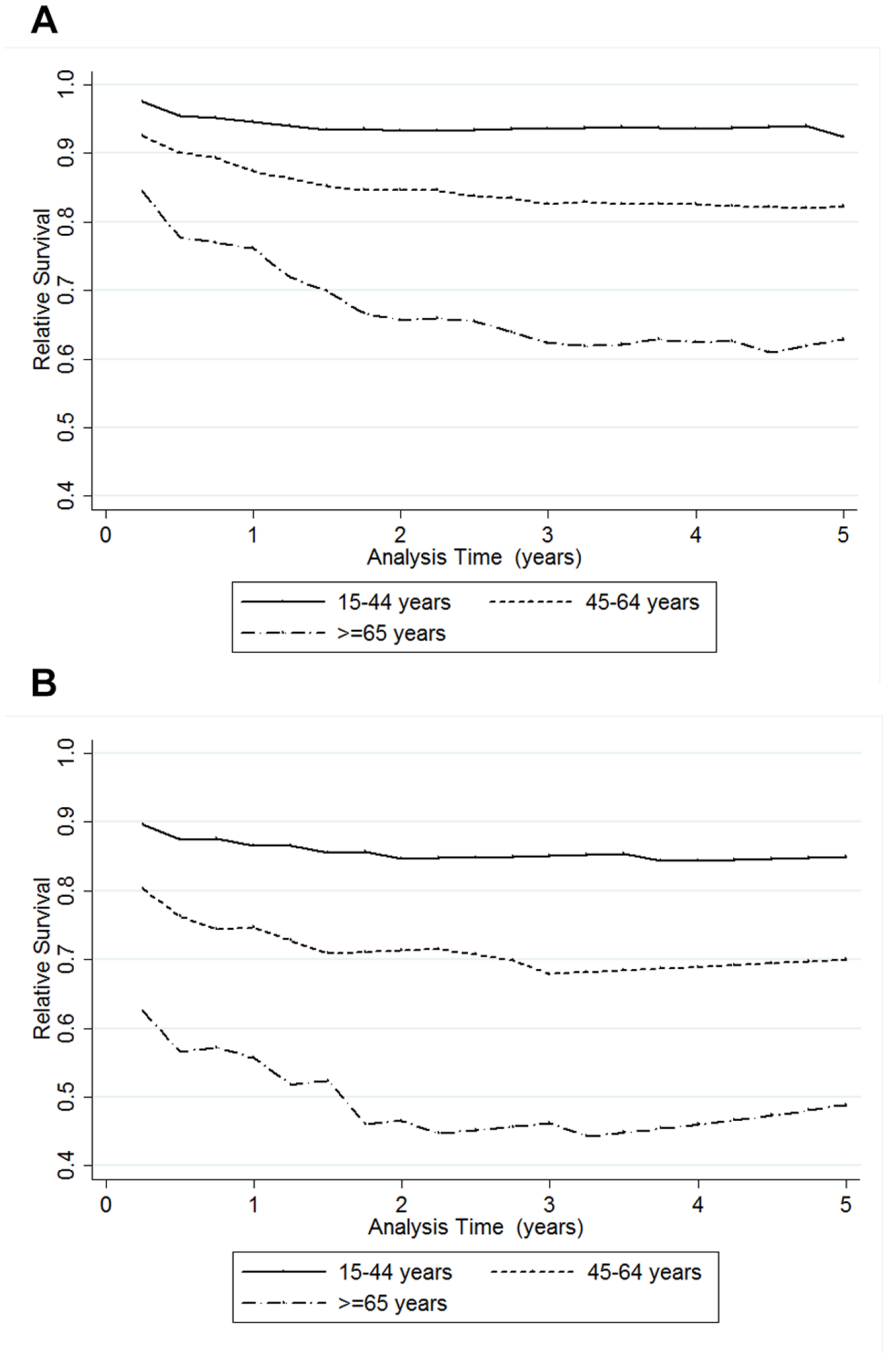
a. Cumulative relative survival by age group for all sepsis patients (n = 1,028). b. Cumulative relative survival by age group for severe sepsis patients (n = 228).

**Figs. 2a** and **2b** show cumulative relative survival by age group for the overall cohort and those with severe sepsis. For the whole cohort, the curve plateaus after approximately 2 years (**Fig. 2a**), and for those with severe sepsis, it plateaus at 3 years (**Fig. 2b**). This indicates that the excess mortality persists for 2 years in sepsis patients, and 3 years in those with severe sepsis, after which sepsis patients have a mortality rate similar to that of the general population. The relative survival of sepsis patients was worse in older age groups, and this difference was most marked in the first 6 months after presentation (**Figs. 3a** and **3b**).

Interval-specific relative survival (**Tables 2** and **3**) lead to a similar conclusion – survival of the sepsis cohort is lower than that of the general population in each period until approximately 2 years after diagnosis, and that of the severe sepsis subgroup until 3 years.

**Table 2:** Interval-specific relative survival by age category (all sepsis patients).

Time period (years of follow up)	Interval-specific relative survival (95% CI)			
	**Overall**	**Age 15–44**	**Age 45–64**	**Age> = 65**
**0–0.5**	0.90 (0.88–0.92)	0.95 (0.93–0.97)	0.90 (0.87–0.93)	0.78 (0.71–0.83)
**0.5–1.0**	0.98 (0.97–0.99)	0.99 (0.98–1.00)	0.97 (0.97–0.99)	0.98 (0.93–1.00)
**1.0–1.5**	0.97 (0.96–0.98)	0.99 (0.97–1.00)	0.97 (0.95–0.99)	0.92 (0.85–0.96)
**1.5–2.0**	0.99 (0.98–1.00)	1.00 (0.98–1.00)	0.99 (0.97–1.00)	0.94 (0.87–0.98)
**2.0–2.5**	1.00 (0.99–1.00)	1.00 (1.00–1.00)	0.99 (0.97–1.00)	1.00 (0.94–1.02)
**2.5–3.0**	0.99 (0.98–1.00)	1.00 (1.00–1.00)	0.99 (0.96–1.00)	0.95 (0.88–0.99)
**3.0–3.5**	1.00 (0.99–1.00)	1.00 (1.00–1.00)	1.00 (0.98–1.00)	1.00 (0.94–1.02)
**3.5–4.0**	1.00 (0.99–1.00)	1.00 (0.98–1.00)	1.00 (0.98–1.00)	1.01 (0.94–1.02)
**4.0–4.5**	1.00 (0.99–1.00)	1.00 (1.00–1.00)	0.99 (0.97–1.00)	0.98 (0.90–1.01)
**4.5–5.0**	1.00 (0.98–1.00)	0.99 (0.96–1.00)	1.00 (0.95–1.01)	1.03 (1.03–1.03)

**Table 3:** Interval specific relative survival by age category (severe sepsis patients).

Time period (years of follow up)	Interval–specific relative survival (95% CI)			
	**Overall**	**Age 15**–**44**	**Age 45**–**64**	**Age> = 65**
**0–0.5**	0.77 (0.70–0.82)	0.87 (0.78–0.93)	0.76 (0.66–0.84)	0.57 (0.41–0.70)
**0.5–1.0**	0.98 (0.95–1.00)	0.99 (0.91–1.00)	0.98 (0.90–1.00)	0.98 (0.77–1.02)
**1.0–1.5**	0.97 (0.92–0.99)	0.99 (0.91–1.00)	0.95 (0.86–0.98)	0.94 (0.73–1.00)
**1.5–2.0**	0.98 (0.94–1.00)	0.99 (0.91–1.00)	1.01 (1.01–1.01)	0.89 (0.66–0.98)
**2.0–2.5**	0.99 (0.96–1.00)	1.00 (1.00–1.00)	0.99 (0.90–1.00)	0.97 (0.71–1.01)
**2.5–3.0**	0.99 (0.95–1.00)	1.00 (1.00–1.00)	0.96 (0.87–0.99)	1.02 (1.02–1.02)
**3.0–3.5**	1.00 (0.96–1.01)	1.00 (1.00–1.00)	1.01 (1.01–1.01)	0.97 (0.70–1.02)
**3.5–4.0**	1.00 (0.96–1.01)	0.99 (0.91–1.00)	1.01 (1.01–1.01)	1.03 (1.03–1.03)
**4.0–4.5**	1.01 (1.01–1.01)	1.00 (1.00–1.00)	1.01 (1.01–1.01)	1.03 (1.03–1.03)
**4.5–5.0**	1.01 (1.01–1.01)	1.00 (1.00–1.00)	1.01 (1.01–1.01)	1.03 (1.03–1.03)

Across all subgroups except males greater than 64 years old, Indigenous patients had lower five-year relative survival than non-Indigenous patients (**Table 4**), with this difference being most evident in young female patients. Across all age groups, females had higher five-year relative survival than males.

**Table 4:** Five-year relative survival (with 95% confidence interval) by age group, sex and Indigenous status.

Gender	Age-group	Non-Indigenous	Indigenous
**Male**	15–44 years	0.98 (0.92–1.00)	0.89 (0.78–0.96)
	45–64 years	0.90 (0.82–0.95)	0.75 (0.63–0.85)
	> = 65 years	0.55 (0.42–0.67)	0.57 (0.26–0.89)
**Female**	15–44 years	1.00 (1.00–1.00)	0.86 (0.77–0.92)
	45–64 years	0.93 (0.83–0.98)	0.74 (0.63–0.83)
	> = 65 years	0.77 (0.54–0.94)	0.72 (0.48–0.92)

### Multivariable analysis of predictors of mortality

In multivariable regression analysis, (**Table 5**) excess mortality was highest in the first year and decreased over time after the sepsis episode, dropping to almost zero (relative to the first year) in the fourth and fifth years of follow-up. Other independent predictors of excess mortality were male sex, Indigenous ethnicity, older age, more comorbidities and sepsis-related organ dysfunction at presentation.

**Table 5:** Multivariable analysis of predictors of excess mortality using Poisson regression.

Variable	Adjusted hazard ratio	95% CI		P-value
**Year of follow-up: 2**	0.35	0.22	0.54	<0.001
**Year of follow-up: 3**	0.12	0.05	0.29	<0.001
**Year of follow-up: 4**	0.04	0.00	0.25	0.001
**Year of follow-up: 5^a^**	0.04	0.00	0.65	0.02
**Male^b^**	1.58	1.10	2.27	0.01
**Indigenous^c^**	1.56	1.05	2.31	0.03
**Age group: 45**–**64^d^**	1.68	1.04	2.73	0.03
**Age group:> = 65^d^**	3.40	2.02	5.72	<0.001
**Charlson index 1**–**2^e^**	3.82	1.90	7.69	<0.001
**Charlson index> = 3^e^**	11.98	6.05	23.74	<0.001
**Severe Sepsis^f^**	2.30	1.61	3.27	<0.001
**Bacteraemic^g^**	1.67	1.15	2.42	0.007

Reference categories – a. First year of follow up; b. Female. c. Non-Indigenous d. Age group 15-44 years. e. Charlson comorbidity index = 0; f. Non-severe sepsis patients; g. Non-bacteraemic patients.

## Discussion

In this cohort of patients hospitalised with sepsis, and including approximately 4,000 patient years of follow up, the excess mortality persisted for 2 years overall and for 3 years in the subgroup with severe sepsis.

Although there is increasing interest in longer term outcomes following sepsis [8]–[11], the use of relative survival analysis is rare in the sepsis literature. Our study differs from the only previous publication to use relative survival analysis in a sepsis cohort [20] in several ways. In contrast to the prospective design of our study, the study by Ghelani was retrospective, with sepsis defined using ICD-9 coding, which decreases both the sensitivity and specificity of the diagnosis of sepsis compared with prospective methods. Ghelani et al found that the cumulative relative survival in the ICU sepsis cohort continued to decline over the entire follow up period (range 4.2–9.6 years), never reaching a plateau. This is in contrast to our finding of a plateau occurring after 2 years, and is difficult to explain. Ghelani's finding implies that the excess risk of death following a sepsis episode never abates, suggesting that the characteristics of the population are responsible rather than the insults of the sepsis episode.

Although we have demonstrated excess mortality in the sepsis cohort compared to the general population, we cannot determine the mechanism of mortality, as we did not have access to cause of death data. Acute sepsis-related organ failure generally resolves by the time of hospital discharge in those who survive. However, systemic inflammatory activation persists for weeks-months afterwards [27], as does functional immunosuppression [28], [29]. Systemic inflammation is associated with endothelial cell activation and dysfunction and an increase in endogenous nitric oxide inhibitors [30]–[33] [34], [35] which are turn associated with acute vascular events [36], [37] and increased early mortality in sepsis [38]. Hence delayed secondary infections and vascular events are plausible potential explanations for this observed excess mortality. An alternative explanation is that patients hospitalised with sepsis may differ from the background population in important comorbidities such as diabetes mellitus, excess alcohol use and chronic renal disease. Our data do not provide evidence to distinguish between these two explanations because the background population life tables used in the relative survival analysis were not stratified by co-morbidity. However, a previous large study by Quartin et al. comparing sepsis patients with a control hospitalised population found that the excess mortality in the sepsis group lasted for at least 5 years, and persisted even after adjustment for comorbidities [13]. This suggests either that the mortality excess which persists beyond the acute period is related to the sepsis episode itself rather than to underlying comorbidities, or that unmeasured comorbidities or characteristics are responsible for excess mortality, as suggested by Ghelani's data [20] Neither our study nor previously published work can definitively determine whether excess mortality in sepsis patients is due to sepsis itself, underlying comorbidities, or a combination of the two.

Our finding that excess mortality persists for at least 2–3 years supports calls to extend the traditional end-points of interventional sepsis studies beyond the most commonly used end-point 28-day mortality [7]. Further work is needed to identify the aetiology of the excess mortality following hospital admission for sepsis, particularly that which occurs more than 1 year post discharge.

Our study has some limitations. We studied a cohort of patients from tropical Australia of whom a substantial proportion were Indigenous Australians, and with a relatively young mean age, and thus the applicability of our findings to other populations is unclear. However, the fact that the post-sepsis mortality excess has been shown to persist for well beyond 90 days in several other cohorts support our findings. We used life tables from 2006 (the most recent available robust data), but the period under study was 2007–2012. If the mortality rate in the background population had decreased over this time, our models may have overestimated the excess mortality in the sepsis population. However, given the short time between these two periods, this is unlikely to have been significant. We did not have access to data on cause of death, nor on comorbidities in the reference population. However, a strength of our study is that, unlike the majority of sepsis studies investigating longer term outcomes post-sepsis [9]–[12], we compared the survival of a sepsis cohort with that of an appropriate reference population.

## Conclusions

In conclusion, the risk of mortality from an acute episode of sepsis extends well beyond hospital discharge. Relative survival analysis is a useful tool for examining the excess mortality in patients with sepsis. Future efforts to improve outcomes from sepsis should focus on endpoints as long as 3 years post discharge, and such endpoints should no longer be limited to the first 90 days following hospital admission.
